# Pathogenicity and transmissibility studies on live attenuated duck enteritis virus vaccine in non-target species

**DOI:** 10.3389/fmicb.2022.979368

**Published:** 2022-11-10

**Authors:** Jie Kong, Keyu Feng, Qiqi Zhao, Yazheng Chen, Jinfeng Wang, Sheng Chen, Guanming Shao, Liqin Liao, Yajuan Li, Zi Xie, Xinheng Zhang, Qingmei Xie

**Affiliations:** ^1^Guangdong Provincial Laboratory of Lingnan Modern Agricultural Science and Technology and Guangdong Provincial Key Lab of Agro-Animal Genomics and Molecular Breeding, College of Animal Science and Veterinary Medicine, South China Agricultural University, Guangzhou, China; ^2^Guangdong Engineering Research Center for Vector Vaccine of Animal Virus, Guangzhou, China; ^3^South China Collaborative Innovation Center for Poultry Disease Control and Product Safety, Guangzhou, China; ^4^Key Laboratory of Animal Health Aquaculture and Environmental Control, Guangzhou, China

**Keywords:** live attenuated duck enteritis virus vaccine, duck enteritis virus, poultry public health, viral transmission, biosafety risks

## Abstract

In the second half of 2021, a highly pathogenic case occurred in a mixed chicken and duck family farm in Guangdong, China. After the duck flocks were immunized with live attenuated duck enteritis virus vaccine (live attenuated DEV vaccine), the chickens of the same farm showed clinical symptoms similar to duck enteritis, such as pericardial effusion, hepatic hemorrhagic spots, kidney enlargement, and intestinal bleeding, with mass mortality. The infection model of target animal tested, as well as the non-target species, was established according to the risk of live attenuated DEV vaccine and transmission in chickens. Live attenuated DEV vaccine was initially replicated in host animals, released the virus, and effectively colonized in the common environment, according to birds challenged experiments. There was evidence to suggest the mode of transmission of duck enteritis virus, and horizontal transmission is the main route of DEV transmission. In addition, high levels of virus titer were detected in chicken embryos and different tissues of SPF chickens. Different degrees of pathological damage occurred in the tissue of chickens. After the SPF chickens were inoculated with live attenuated DEV vaccine, different degrees of virulence were exhibited, pointing to a potential risk to other domestic bird species.

## Introduction

Ducks play an important role in the host of duck enteritis virus (DEV) in nature, and the successful control of epidemic disease in ducks has important implications for the circulation of the disease and its prevention in waterfowl. Despite substantial effortsto control virus transmission, DEV has continued to infect and spread, indicating that the threat they pose to both domestic poultry and public health has not diminished. To date, DEV has been reported in France, the United States, Canada, and other countries successively, and it has resulted in significant economic losses in modern farming (Hall and Simmons, 1972; Hanson and Willis, 1976; Zhang et al., 2020; Chen et al., 2021; Islam et al., 2021; Shen et al., 2021). In China, thousands of ducks are reared annually in open fields, with no biosecurity measures in place. Since the 1960s, researchers have been working on live attenuated DEV vaccine, and billions of doses have been administered there annually on duck farms. Live attenuated DEV vaccine was used routinely to control lethal DEV infection in many duck-producing areas.

In 1940, scholars first proposed the term “duck plague.” Duck plague virus (DPV) was declared a duck epidemic disease by the International Veterinary Society in 1949 after confirmation (Leibovitz and Hwang, 1968; Gough and Alexander, 1990; Pearson and Cassidy, 1997). DEV pathogen in domestic ducks has been documented, and some of the strains responsible for these outbreaks are lethal to ducks in the research setting, but it is not known whether DEV strain replicates in chickens symptomatically or asymptomatically. After the duck flocks were infected with DEV strain, it showed typical clinical symptoms, such as vascular and digestive mucosal damage, tissue hemorrhage, severe diarrhea, and degenerative lesions in parenchymatous tissue. Herpesvirus family encompasses a wide range of natural pathogens that it used numerous virulence factors for pathogenicity and fitness in non-target species. Not only in the target species but also in the non-target species, it is important to determine possible pathogenicity, excretion, and transmission of the vaccine virus. Despite the live attenuated DEV vaccine has been used as an effective control tool to prevent DEV transmission, no potential threat to other animals has been reported (Huang et al., 2014). Therefore, assessing of the emerging infectivity and pathogenicity of animal pathogens or biologics infecting different hosts is essential for understanding the epidemiology and preventive measures.

In 2021, a case characterized by sudden onset was observed in a mixed chicken and duck family farm in Yunfu, Guangdong Province, China. After the chickens were affected in the mixed farm, it showed marked depression, with mass mortality. After confirmation, it was determined that the farm’s ducks were immunized with live attenuated DEV vaccine, and the chickens exhibited acute, contagious, and septic symptoms. To determine the risk of active and passive transmission of live attenuated DEV vaccine to chickens, we restored the original breeding state of target ducks immunized. We placed specific-pathogen-free (SPF) chickens in duck flocks normally immunized with live attenuated DEV vaccine. Birds were characterized by clinical symptoms and molecular biology detection to observe pathogenicity and estimate the biosafety risks. At the same time, we treated the flock with manual injections after anesthetization to observe whether the birds had different infection states under passive infection and active infection. The objective of this study was to determine whether live attenuated DEV vaccine developed clinical signs in chickens and whether it was able to propagate and shed the virus after exposure to chickens thus having potential breeding risks for the bird industry.

## Materials and methods

### Virus, vaccine, and animals

DEV AV1221 strain (GenBank: EF173464.1) and live attenuated DEV vaccine were obtained from the China Veterinary Culture Collection (CVCC), and DEV AV1221 was propagated in primary duck embryo fibroblasts (DEFs). Specific-pathogen-free (SPF) chickens and Specific-pathogen-free (SPF) chicken embryos were obtained from Xinxing DHN Egg Co., Ltd., Guangdong. One-day-old ducklings free of duck plague virus-specific maternal antibody were obtained from the WENS, Guangdong, China. All studies described here were approved by the Research Ethics Committee and Institutional Animal Care and Use Committee of South China Agricultural University.

### Pathogenicity of live attenuated duck enteritis virus vaccine in SPF-chicken embryo

To evaluate the virulence of the live attenuated DEV vaccine in SPF-chicken embryos, four groups (10 replicates per group) of forty SPF-chicken embryos were inoculated with 0.1 ml volume of 10^4^.^00^ or 10^5^.^00^ median embryo lethal dose (ELD_50_) of vaccine by two routes [Chorioallantoic membrane (CAM) or allantoic cavity; Berhane et al., 2021]. Two groups of SPF-chicken embryos were inoculated with PBS without virus as a parallel negative control, and another two groups of SPF-chicken embryos were inoculated with 10^5^.^42^ tissue culture infectious dose (TCID_50_) of DEV AV1221 as a parallel positive control. The challenged and control group were separately housed in separate incubators, and the embryos were candled daily to monitor for mortality while the lesions and mortality of the embryos were recorded. Total genome was extracted from the embryo or CAM to check the presence of DEV using the specific primer (F: 5′-GAACTGAGCGATATGATAG-3′, R: 5′-CGACTGATGACAATGAAT-3′).

### Transmission studies of live attenuated duck enteritis virus vaccine in mixed farming flocks

To detect pathogenicity and transmission in chickens *in vivo*, eighty, 1-week-old ducks were randomly divided into four groups of twenty ducks. For virus contact transmission studies, groups of ducks were anesthetized with CO_2_ and infected with corresponding DEV AV1221 at a dose of 10^5^.^42^ TCID_50_ in 0.1 ml volume, respectively. At 1-day postinoculation (dpi), twenty chickens were contacted together with the challenged ducks (Yu et al., 2021).

For vaccine contact transmission studies (Karlsson et al., 2014), groups of ducks were anesthetized with CO_2_ and inoculated intramuscularly live attenuated DEV vaccine with 10^4^.^00^ ELD_50_ or 10^5^.^00^ ELD_50_ in 0.1 ml volume. One dpi later, chickens (*n* = 20 per each group) were placed in separate negative pressure isolators with the inoculated group (Zhu et al., 2013). Body status and healthy check were assessed every day. For virus contact transmission studies and vaccine contact transmission studies, three infected ducks, three physical contact chickens, and three parallel control animals were euthanized. The remaining ducks or chickens at the end of the study were euthanized by the intravenous administration of sodium pentobarbital (100 mg/kg body weight).

### Genome extraction and real-time polymerase chain reaction verification

Animals from each group were euthanized and necropsied. Tissues (including liver, heart, spleen, glandular stomach, kidney, and intestine) were fixed in 4% paraformaldehyde. Half of the samples were embedded in paraffin, sectioned, and stained with hematoxylin–eosin (H&E) (Gautier, 2017). A portion of samples (500 mg) was taken out from low temperature refrigerator (−20°C), and each tissue was homogenized in 2.0 mL pre-cooled phosphate-buffered saline (PBS). All of them were extracted by SteadyPure Viral DNA/RNA Kit and stored at −80°C, and PCR amplification was performed with a pair of particular primers (F:5′-GAACTGAGCGATATGATAG-3′, R:5′-CGACTGATGACAATGAAT-3′). To test virus titer, the DEV AV1221 genome was utilized as a template, and the recycling product was ligated with the pMD-19T vector to create a standard plasmid as the standard curve. Genome of tissue (including liver, heart, spleen, glandular stomach, kidney, and intestine) was extracted by SteadyPure Viral DNA/RNA Kit for real-time fluorescence quantitative PCR (Yu et al., 2020). PCR reaction conditions were as follows: 95°C for 30 s, 34 cycles of amplification at 95°C for 10 s, and 60°C for 30 s, followed by a dissociation curve analysis step.

### Environmental sample collection

Tissue was taken during the tested period, and they were always done in mixed groups. Appropriate soil and sink (Zani et al., 2020) were sampled superficially from an area of isolators using a 2 ml Eppendorf tube, and 1 ml of 0.067 M phosphate buffer, pH 7.5, or 250 μl of 1-mm-diameter sterile glass beads were added to each tube (Eisen et al., 2019). Samples were transported to the Laboratory of Poultry Research and promptly processed for molecular biology detection, and aliquots of samples were maintained in an ultra-low temperature refrigerator (−80°C) for viral genome detection. Samples were extracted by SteadyPure Viral DNA/RNA Kit, and it was used for real-time fluorescence quantitative PCR.

### Pathogenicity of live attenuated duck enteritis virus vaccine to chickens

To characterize the pathogenicity of live attenuated DEV vaccine currently using immunization program, SPF chickens were chosen for active infection experiment. Eighty, 1-day-old chickens with negative sera against duck enteritis virus were randomly separated into four groups, and chickens were maintained in isolators with negative pressure, with free access to food and water. Forty chickens were inoculated with live attenuated DEV vaccine at a dose of 10^4.00^ ELD_50_ or 10^5.00^ ELD_50_ in 0.1 ml volume, as the challenged group, after chickens were anesthetized with CO_2_. Twenty chickens were inoculated at a dose of 10^5.42^ TCID_50_ in 0.1 ml volume as the infected group. Others were inoculated with PBS in the same isolators as the negative control group. Until 10 dpi, chicken signs were detected. Euthanized animals in advance if they appeared mental depression. The remaining ducks or chickens at the end of the study were euthanized by the intravenous administration of sodium pentobarbital (100 mg/kg body weight).

### Statistical analysis

Data analysis was conducted using GraphPad Prism 8.0. Two-tailed Student’s t-test was used to determine statistical significance (Zhou et al., 2020), and *P* < *0.05* was considered statistically significant.

## Results

### Chicken embryos developed simultaneously pathogenicity or death

**FIGURE 1:**
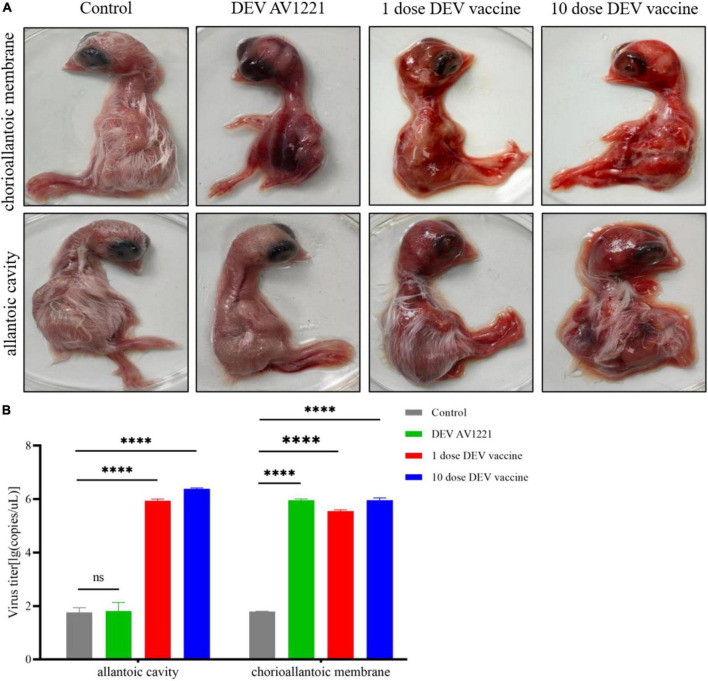
Replicative capacity of live attenuated duck enteritis virus (DEV) vaccine to chicken embryos *in vivo*. **(A)** Pathogenicity and gross damage of live attenuated DEV vaccine in chicken embryos. **(B)** Virus replication of live attenuated DEV vaccine in chicken embryos. SPF-chicken embryos, 1-day-old, were randomly divided into four groups, and each group covers ten chicken embryos. From left to right being challenged with DEV AV1221 (10^5^.^42^ TCID_50_), 1 dose of vaccine (10^3^.^00^ ELD_50_), and 10 dose of vaccine (10^4^.^00^ ELD_50_), at the same dose in 100 μL, respectively. The results are representative of two tests. ^****^P < 0.0001.

**FIGURE 2:**
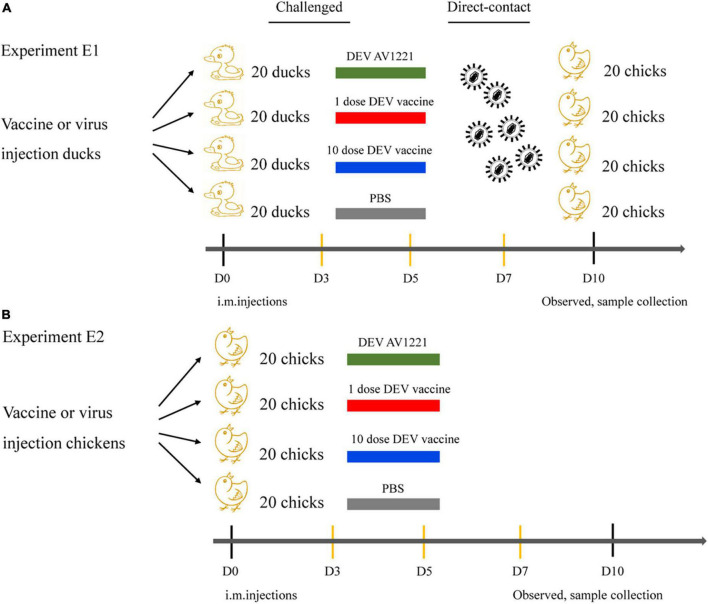
Diagram of animal regression experiments. **(A)** Ducks were randomly divided into four groups of twenty ducks. A quarter of ducks were infected with corresponding DEV AV1221 at a dose of 10^5.42^ TCID_50_ in 0.1ml volume. A quarter of ducks were used as negative controls. The rest of the ducks was equally divided into two groups, half ducks were immunized with live attenuated DEV vaccine of 10^4.00^ ELD_50_ in 0.1 ml volume, and the other half of ducks were immunized with the same vaccine of 10^5.00^ ELD_50_ in 0.1 ml volume. SPF chickens were contacted with the challenged ducks in the same isolator, respectively. **(B)** Chickens were randomly divided into four groups of twenty chickens. A quarter of chickens were infected with corresponding DEV AV1221 at a dose of 10^5.42^ TCID_50_ in 0.1ml volume. A quarter of chickens were used as negative controls. The rest of the chickens were equally divided into two groups, half chickens were immunized with live attenuated DEV vaccine of 10^4.00^ ELD_50_ in 0.1 ml volume, and the other half of chickens were immunized with the same vaccine of 10^5.00^ ELD_50_ in 0.1 ml volume. SPF chickens were directly challenged.

Based on differences in inoculation route specificity among the duck enteritis virus, chicken embryos were inoculated with live attenuated DEV vaccine at a dose of 10^4.00^ or 10^5.00^ ELD_50_ and 10^5.42^ TCID_50_ units of DEV AV1221. One dpi later, the embryos were candled to monitor for mortality. Embryos were monitored for death signs during 4 to 5 dpi. Intriguingly, chicken embryos were exposed to live attenuated DEV vaccine in two routes at the same time, resulting in growth retardation and severe widespread bleeding (Figure 1A). Most critically, when the embryos were exposed to the live attenuated DEV vaccine, they all died. Chicken embryos were inoculated with DEV AV1221 through the CAM route and died, compared with the allantoic cavity route. While there was no evidence of embryonic death in DEV AV1221 infection *via* the allantoic cavity route, embryos implanted with the virus showed overt clinical indications at 3 to 5 dpi (Figure 1A). Live attenuated DEV vaccine replicated well in chicken embryos, the CAM route was favored as the virus replicates well in chorioallantoic membrane, and high virus titer was found in these membranes and associated embryo (Glavits et al., 1990). The virus titer in tissue collected from different groups varied, indicating that both DEV and live attenuated DEV vaccine were pathogenic or deadly to chicken embryos. Diagram legends provide the animal designs of each group (Figure 2, Table 1), and virus titer of each group is shown in Figure 1B.

**TABLE 1:** Record of the animal regression experiments.

Hosts	Groups	Vaccine	Numbers	Day	Dose/0.1 ml	Number of hosts to death in each day of age/day post-inoculation										% Mortality
						1	2	3	4	5	6	7	8	9	10	
Duck	Challenged	DEV AV1221	20	7	10^5.42^TCID_50_	0	2	8	7	3	/	/	/	/	/	100
Duck	Challenged	1 dose DEV vaccine	20	7	10^4.00^ELD_50_	0	0	0	0	0	0	0	0	0	0	0
Duck	Challenged	10 dose DEV vaccine	20	7	10^5.00^ELD_50_	0	0	0	0	0	0	0	0	0	0	0
Duck	Challenged	Control	20	7	PBS	0	0	0	0	0	0	0	0	0	0	0
Chicken	Direct-contact	/	20	1	/	0	0	0	0	0	0	0	0	0	0	0
Chicken	Direct-contact	/	20	1	/	0	0	0	2	1	/	/	/	/	/	15
Chicken	Direct-contact	/	20	1	/	0	0	2	4	3	/	/	/	/	/	45
Chicken	Direct-contact	/	20	1	/	0	0	0	0	0	0	0	0	0	0	0
Chicken	Challenged	DEV AV1221	20	1	10^5.42^TCID_50_	0	0	0	0	0	0	6	2	0	0	40
Chicken	Challenged	1 dose DEV vaccine	20	1	10^4.00^ELD_50_	0	0	0	16	4	/	/	/	/	/	100
Chicken	Challenged	10 dose DEV vaccine	20	1	10^5.00^ELD_50_	0	0	6	14	/	/	/	/	/	/	100
Chicken	Challenged	Control	20	1	PBS	0	0	0	0	0	0	0	0	0	0	0

### Pathogenicity lesions of ducks and chickens in transmission studies

**FIGURE 3:**
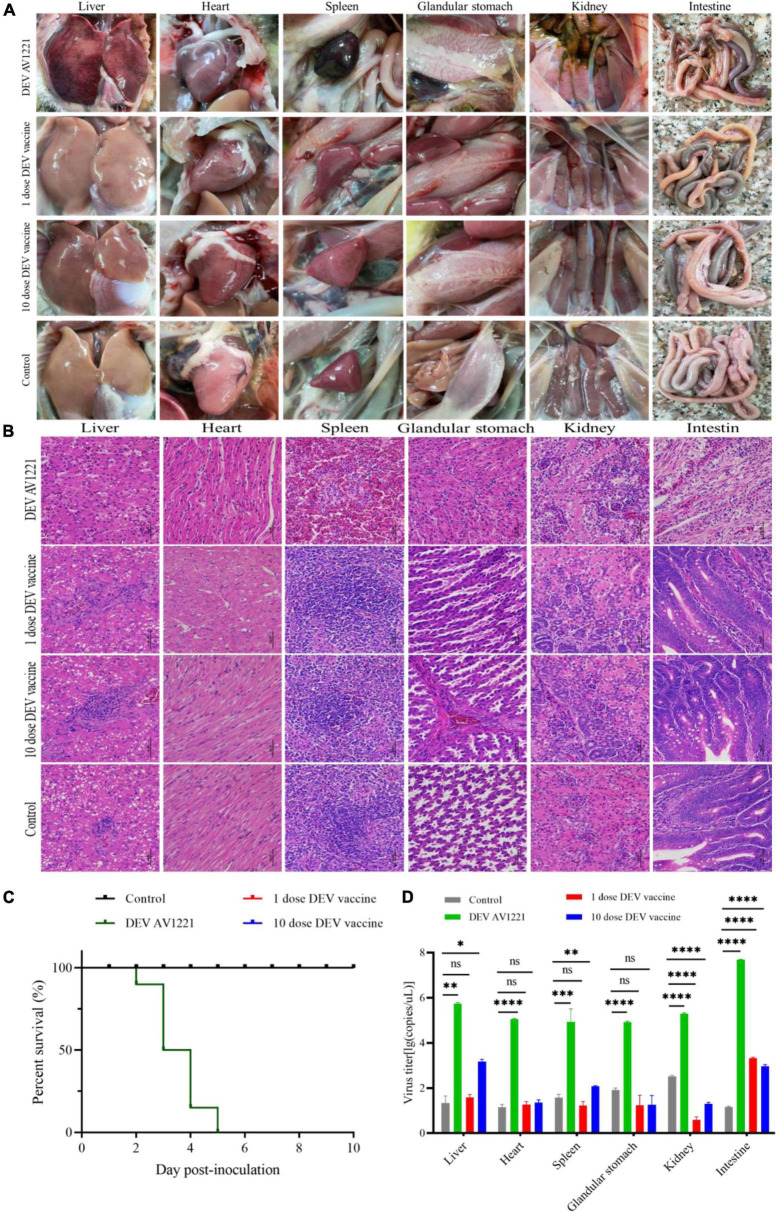
Live attenuated duck enteritis virus (DEV) vaccine in targeted ducks retains *in vivo* growth kinetics activity. **(A)** Gross lesions of target ducks. **(B)** The hematoxylin- and eosin (H&E)-stained tissue section in target ducks. From left to right are liver, heart, spleen, glandular stomach, liver, kidney, and intestine. **(C)** The survival curve of target ducks. **(D)** DEV effectively replicates in different tissue of target ducks. Data are representative of four experiments related to challenge. ^**^P < 0.01, ^***^P < 0.001, and ^****^P < 0.0001.

**FIGURE 4:**
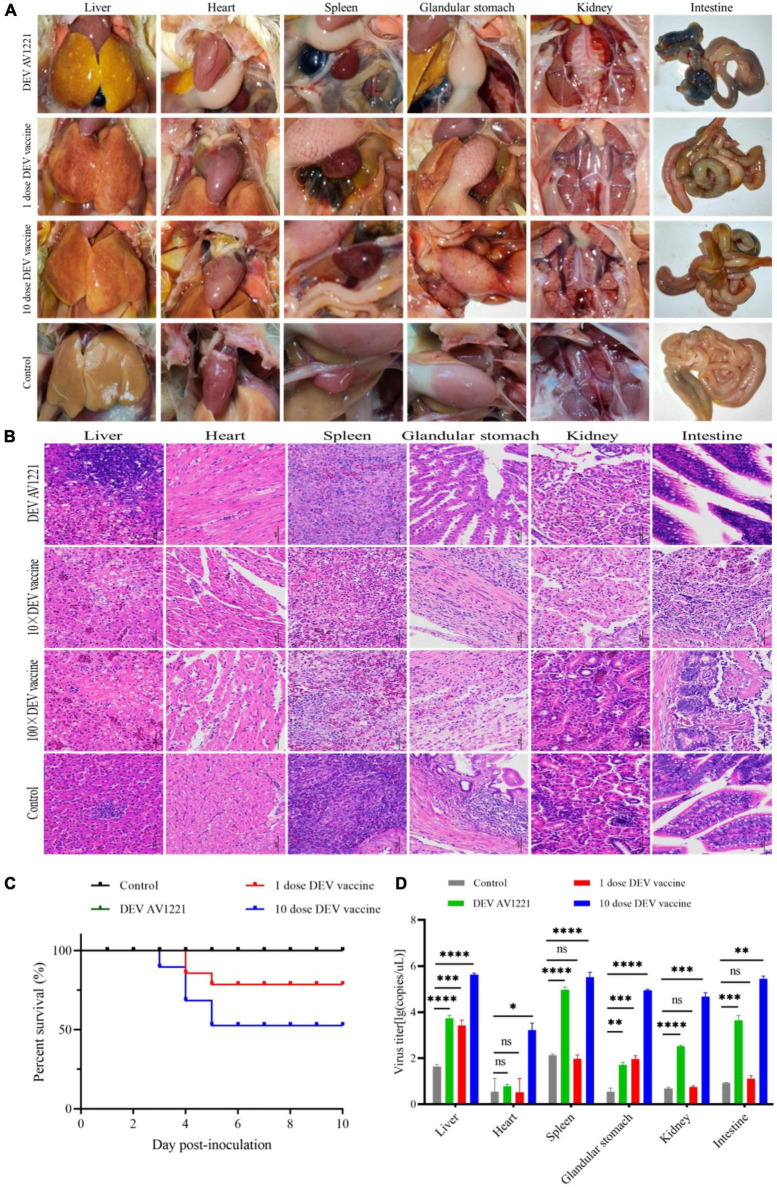
Live attenuated duck enteritis virus (DEV) vaccine replicated in non-target chickens and resulted in effectively virus transmission. **(A)** Pathogenic lesions of non-target chickens. **(B)** The hematoxylin- and eosin (H&E)-stained tissue section in non-target chickens. From left to right are liver, heart, spleen, glandular stomach, liver, kidney, and intestine. **(C)** The survival curve of non-target chickens. **(D)** DEV effectively replicates in different tissue of non-target chickens. Data are representative of four experiments related to challenge. ^**^P < 0.01, ^***^P < 0.001, and ^****^P < 0.0001.

Given that transmission studies surround DEV AV1221 and live attenuated DEV vaccine, clinical symptom changes were performed and revealed that pathogenicity lesions of target ducks and non-target chickens revealed that the vaccine had stronger affinity and transmission for susceptible chickens than DEV AV1221. Although no obvious clinical signs of disease were observed after chickens were exposed to ducks were infected with DEV AV1221, significant lesions were observed after chickens exposed to ducks were vaccinated with live attenuated DEV vaccine, and 10 doses of vaccine caused more serious complications (Figures 3A, 4A). Severe liver bleeding, pericardial effusion, splenomegaly, and hemorrhagic necrosis were discovered during the autopsy, as well as other consistent signs, such as acute anorexia, diarrhea, sadness, and paralysis. At 10 dpi, negative control chickens were euthanized with no significant histological lesions or death, further confirming the lesions in the challenged groups. In summation, these findings enriched animal research demonstrating that chickens occurred pathogenicity and lesions after contacting ducks.

### Histopathology observation of transmission studies

The next step was to see whether the inoculation of duck enteritis virus or vaccine enhances lesions against ducks and chickens. Initially, we collected duck tissue from the liver, heart, spleen, glandular stomach, kidney, and intestine, which are all susceptible internal tissue. During the experimental infection, the control group had no microscopic lesions. Viruses enhanced the tissue damage in the infection groups, in contrast to the negative control. Analysis indicated necrosis of liver cell necrosis and hemorrhage. Inflammatory cell infiltration, localized edema, and loosely organized myocardial fibers were seen in the heart. The vascularity of certain marginal myocardial interstitium appeared sluggish and dilated. The spleen had extensive erythrocyte proliferation and disordered tissue structure, as well as many sites of necrosis. Capillary bruising was seen in the glandular stomach, and a large number of epithelial cells in the mucosal layer were lost or disappeared. A limited amount of intrafollicular glandular damage, structural destruction, and lysis accompanied the submucosal layer. Tubular atrophy and moderate dilation of the renal capsule were seen in the kidneys. There was significant tubular epithelial cell necrosis and detachment, minor interstitial bruising, and dilatation of the renal tubules on histological examination. There was massive villi breakage and shedding in the intestinal mucosal layer. A large number of epithelial cells were shed, and some capillaries became stagnant and dilated (Figure 3B). Compared to the infection group, either 1 dose of vaccine or 10 doses of vaccine enhanced portion of lesions in ducks, with the main lesions occurring in the liver, spleen, and intestine (Figure 3B).

To monitor the histopathology of SPF chickens, we collected concurrently chickens’ samples which are common tissue, including liver, heart, spleen, glandular stomach, kidney, and intestine at 1 dpi. Clear histopathological lesions were found in chickens who encountered ducks that had received 1 dose or 10 doses of the live attenuated DEV vaccine. Hepatocytes that were missing some of cytoplasm only showed eosinophilic change, widespread vacuolar degeneration, and numerous localized necrosis. Infiltration of inflammatory cells happened in the heart. Increased macrophages, heightened eosinophilia, nuclear fragmentation or consolidation of spleen cells around spleen necrotic foci, intestinal cells autolysis, and the nucleus were solidified (Figure 4B). Only the live attenuated DEV vaccine induced a major histological disorder. Compared with the inoculation of 1 dose or 10 doses of live attenuated DEV vaccine, DEV does not cause the significant histopathological disorder.

### Clinical status observation

The changes in lifespan in response to survival curve were reflected by distinctive changes in survival rate decreased over time. We next examined the respond of virus infection and live attenuated DEV vaccine inoculation in ducks and chickens following experiment period. After the ducks were infected, DEV showed clinical symptoms, with mass mortality (Figure 3C). Remarkably, chickens exposed to the diseased ducks showed no clinical symptoms. In the days following infection, the survival rate was associated with different doses of live attenuated DEV vaccine. Different doses of live attenuated DEV vaccine caused harm to both the directly contacted chickens and the systemically challenged ducks. Interestingly, virus could not transmit among ducks to chickens through physical contact, while live attenuated DEV vaccine could transmit to the chickens, even leading to death. Another study examining direct contact transmission (by contacting with ducks were infected with DEV) found lower transmission rates: no chickens occurred death. In contrast, direct contact transmission (by contacting with ducks were immunized with live attenuated DEV vaccine) resulted in the infection of 15 to 45% of susceptible chickens (Figure 4C). On another isolator, the latter test was conducted. Together, the research showed that chicken occurred death after exposure to live attenuated DEV vaccine of ducks.

### Virus efficiently replicated in target and non-target species

Replication was evaluated in different tissues to further characterize the virulence and titer of tissue in ducks and mixed farmed chickens. Under normal conditions, an increase in viral titer would be observed if the virus replicates in the tissue. Briefly, different levels of virus titer were detected in all tissue collected from birds through real-time PCR verification. Viruses associated with infected ducks replicated faster in all tissue studied. In comparison with DEV AV1221 and the negative control group, birds with immunized with live attenuated DEV vaccine increased titers and replicated to higher titers overall (Figures 3D, 4D). The number of DEV copies replicated well in the liver of chickens. Moreover, the overall number of virus copies was higher in the 10 dose of live attenuated DEV vaccine than in the 1 dose of live attenuated DEV at the tested tissue. Although not as striking, the replication kinetics was more rapid in liver, spleen, and intestine than in other tissue, DEV and live attenuated DEV vaccine could replicate in the non-target species.

### Vaccine has efficient contact transmission and deposition on surfaces

**FIGURE 5:**
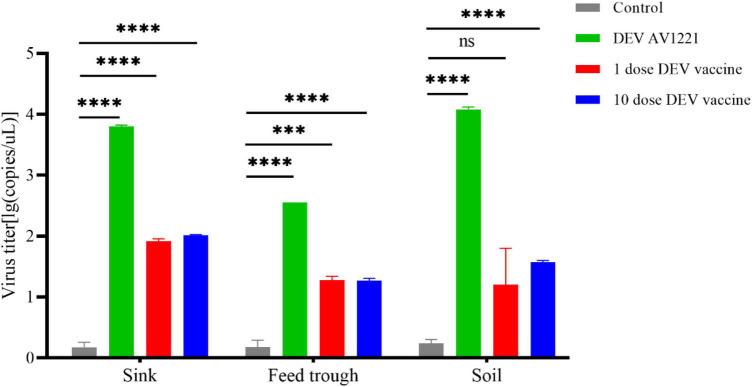
Duck enteritis virus (DEV) replicated and resulted in effectively virus shedding. During the target ducks-to-non-target chicken transmission, the virions shed in sink, soil, and feed trough may persist for long periods in the environment. Virus titer was expressed as lg(copies/μl). ^***^P < 0.001, ****P < 0.0001.

In general, the persistence results demonstrated that DEV may survive for days to weeks, depending on the soil, sink, and feed trough. Contact transmission of vaccine virus between ducks and chickens appeared affected by environmental factors. In these trials, ducks and non-target chickens were maintained in the same isolators, so it is plausible to conclude that the transmission was by direct contact. The highest persistence of virus was found in the soil, followed by the sink (Figure 5), and the vaccine virus was found at relatively low levels in environmental samples (virus titer was detected on any environmental materials). The durability of the live attenuated DEV vaccine and DEV was demonstrated in these investigations.

### Virus and related vaccine replicate efficiently *in vivo* of chickens

**FIGURE 6:**
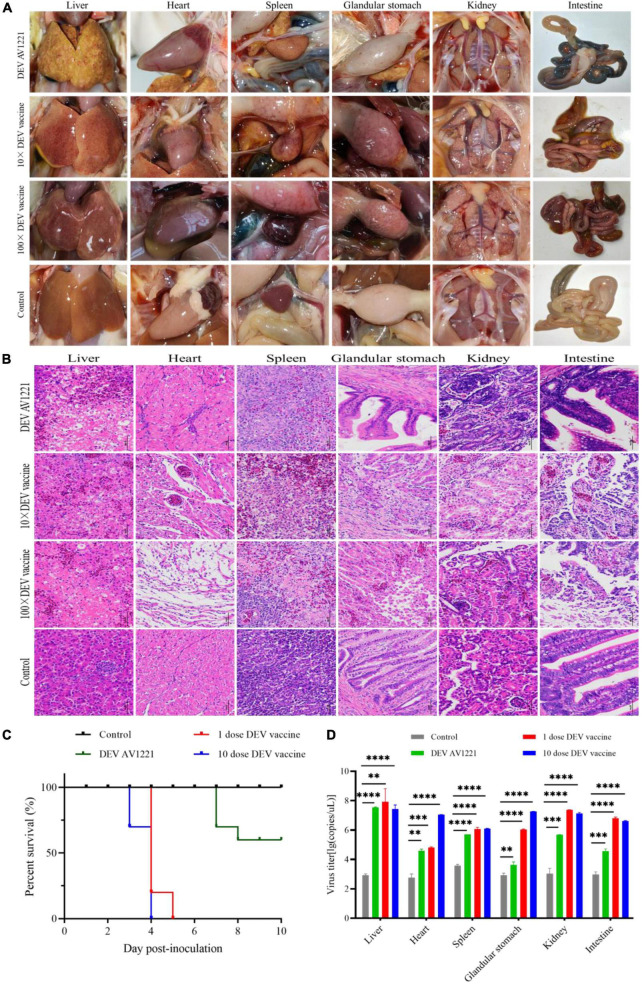
Both duck enteritis virus (DEV) and Live attenuated DEV vaccine replicated directly in targeted chickens and resulted in tissue damage. **(A)** Serious lesions of target chickens. **(B)** The hematoxylin- and eosin (H&E)-stained tissue section in target chickens. From left to right are liver, heart, spleen, glandular stomach, liver, kidney, and intestine. **(C)** The survival curve of target chickens. **(D)** DEV effectively replicates in different tissue of target chickens. Data are representative of four experiments related to challenge. ^**^P < 0.01, ^***^P < 0.001, ^****^P < 0.0001.

Finally, to observe pathogenicity, one group of chickens was directly infected DEV at a dose of 10^5.42^ TCID_50_ units, and other groups were immunized with live attenuated DEV vaccine at a dose of 10^4.00^ ELD_50_ or 10^5.00^ ELD_50_, respectively, and clinical status was monitored during the experiment period (Figure 6A). Histopathology observation and virus titer were performed as described above (Figure 6B).

Similar to previous direct contact tests, all of the chickens were directly challenged. Meanwhile, both DEV and live attenuated DEV vaccine could cause significant pathogenicity lesions and clinical death compared with the control group. Chickens were immunized with a dose of 10^4.00^ ELD_50_ or 10^5.00^ ELD_50_, and all of chickens (*n* = 40) were infected (Figure 6C). Based on qPCR results, high levels of virus titer were detected in all tissues collected from chickens using different inoculation doses. The fact indicated that DEV and live attenuated DEV vaccine were capable of affecting chickens and were extremely susceptible to different tissue (Figure 6D). In contrast, the replication kinetics of live attenuated DEV vaccine was more rapid in the tissue, and high levels of virus titer could be detected.

## Discussion

As a highly pathogenic and infectious pathogen in waterfowl, duck enteritis virus has been well documented over the last decades (Goldberg et al., 1990; Li et al., 2016). Live attenuated DEV vaccines have been developed and used in different countries to control lethal damage in ducks (Huang et al., 2014). A live attenuated DEV vaccine was first studied by Jansen, 1964, who lowered the virulence of DEV by exposing it through multiple chicken embryos. Their study found that inoculated ducks could be protected against DEV challenge (Dardiri, 1975). Live attenuated DEV vaccine virus used and tested in this study was derived by passaging the lethal DEV in CEFs and has been used in the field since the 1960s (Aravind et al., 2015). To data, a commercial live attenuated DEV vaccine effectively controlled the disease (Mondal et al., 2010; Zou et al., 2015; Yang et al., 2021), but no study has ever evaluated whether immunization posed risks to non-target species in the mixed flocks. For the first time, we report on the morbidity symptoms in chickens linked with transmission, and DEV and corresponding live attenuated DEV vaccine have been assessed for pathogenicity and risks in chickens. These findings imply that there are existing facts of live attenuated DEV vaccine transmission from target ducks to non-target chickens.

To further investigate the pathogenicity of live attenuated DEV vaccine, we successfully conducted animal regression experiments using SPF chickens. In comparison with the control group, pathogenicity and histopathology data showed that chicken growth was hindered and compromised. Chickens challenged with DEV or live attenuated DEV vaccine have the most pronounced influence on growth within 3 to 5 days postinoculation. It was indicated that chickens exposed to DEV or the live attenuated DEV vaccine caused death. Chickens challenged by live attenuated DEV vaccine showed similar signs, such as liver hemorrhage, spleen necrosis, and intestinal congestion and hemorrhage, consistent with ducks that were infected DEV strain. Unlike previous reports, this study surprisingly revealed that chickens immunized with live attenuated DEV vaccine showed glandular hemorrhage, pericardial effusion, swollen and congested kidneys, and enhanced histological damage, even leading to death. Interestingly, after ducks were immunized with live attenuated DEV vaccine, chickens are infected through direct contact, but ducks were infected with DEV, and chickens in the same flock were not significantly affected. Here, we report that high viral titer and pathogenic damage observed in chickens were inoculated with live attenuated DEV vaccine.

Commercial vaccines, especially live attenuated DEV vaccine, can easily be transmitted when circulating or being used in the farms. A live attenuated DEV vaccine has been developed and utilized to control duck enteritis virus for many years (Zou et al., 2015). However, DEV has a broad tropism and can establish latency in the trigeminal ganglia, lymphoid tissues, and peripheral blood lymphocytes, in which they efficiently induce both humoral immune and cellular immune responses (Shawky and Schat, 2002; Jiang et al., 2007). Meanwhile, DEV can be transmitted by direct contact or indirectly through environmental contamination (Kaleta et al., 2007; Li et al., 2016). Studies have shown that duck flocks’ immunization with live attenuated DEV vaccine can prevent the occurrence of pathogens, but after immunization, it is impossible to distinguish whether it is wild virus infection or vaccine immunization. It is further speculated whether immunization with live attenuated DEV vaccine will cause a risk of transmitting the duck enteritis virus (Dhama et al., 2017; Ning et al., 2022). In Guangdong Province, where mixed farms and small-scale family farms are common, it is possible for farm birds in the same flock to contract several pathogens through cross-host transmission. Here, we tested and evaluated a viral transmission phenomenon depending on mixed bird farms. Given its circulation in non-target species, the detected results raise concerns about the advent of this species as a highly dangerous and contagious illness. Biosafety concerns arose from the use of a domestic live attenuated DEV vaccine. These findings are critical in determining the safety of birdrearing.

## Data availability statement

The original contributions presented in the study are included in the article/supplementary material, further inquiries can be directed to the corresponding author.

## Ethics statement

The animal study was reviewed and approved by South China Agricultural University Committee of Animal Experiments approval ID: SYXK2019-0136. Written informed consent was obtained from the owners for the participation of their animals in this study.

## Author contributions

JK designed the project and finalized the manuscript. KF, QZ, and LL performed the data analysis. YC and JW performed the experiments. SC and GS performed the supervision. ZX and XZ conducted the validation. All authors contributed to the article and approved the submitted version.
